# High-performance ceramic/epoxy composite adhesives enabled by rational ceramic bandgaps

**DOI:** 10.1038/s41598-019-57074-7

**Published:** 2020-01-16

**Authors:** J. B. Hu

**Affiliations:** School of Robot Engineering, Yangtze Normal University, Chongqing, 408100 P.R. China

**Keywords:** Energy science and technology, Energy science and technology, Materials science, Materials science, Materials science

## Abstract

High over-all properties, including low dielectric loss, high breakdown strength, high mechanical shock strength, high thermal conductivity and high weight stability, are very difficult to simultaneously achieve in electrical-insulation applicable cured potting-adhesive materials. To deal with this challenge, in this work, we have designed and fabricated a series of epoxy based composite potting-adhesives filled with low-cost and high-performance inorganic micro-particles including alpha-silica, alpha-alumina and alpha-SiC. Combination employment of high-molecular-weight and low-molecular-weight epoxy resins as matrices has been made. Heat-induced curing or crosslink of resin matrices has been carried out. Large band gap of silica filler has endowed the cured composite with high breakdown strength and ageing breakdown strength, and meanwhile relatively high deformation trait of silica has led to high shock strength of cured composite. Silica filler has been found to be superior to other two fillers, namely, optimal over-all properties such as dielectric, breakdown, mechanical and thermal features have been obtained in silica filled cured composite. High breakdown strength of ~48 MV m^−1^ and shock strength of ~9950 J m^−2^ have been achieved in silica loaded composite. This work might open up the way for large-scale fabrication of promising epoxy-based hybrid potting-adhesives.

## Introduction

Recently, potting adhesive materials with promising electrical, mechanical and thermal properties have triggered general attention and research interest in the field of fabricating aerospace applicable special power supplies^1^. Inorganic particles filled polymer based hybrid potting adhesives are consisting of polymer matrix, curing-agent, curing boosting agent, plasticizer, thermally conductive filler, reactive diluent and demoulding agent^2^. Plenty of epoxy resins in different trademarks have been employed as matrix materials of hybrid potting adhesives, thanks to their high Young’s modulus, strong bonding force toward massive substrates, high solvent resistance and low production cost. Mixing different epoxy resins as matrices has been found to be highly helpful to achieving desirable high fluidity and high mechanical strength in composite potting adhesives, at the same time^3^. Furthermore, lots of acid-anhydride derived curing-agents such as methyl tetrahydrophthalic anhydride have been adopted in hybrid potting adhesives, attributed to rather wide temperature-applying range and favorable high electric insulation trait of cured epoxy based adhesives gained via those curing-agents^4^. By using those acid-anhydride derived curing-agents, potting adhesives have achieved several desired features such as easy perfusion process, low curing volume-shrinkage and high curing mechanical property. Besides, amine derived curing-agents have been found to result in property failure of cured adhesives at high temperature, but no performance failure was found at low temperature^5^.

Although huge success has been obtained in acid-anhydride derived curing agents, the undesired high curing temperature of those curing agents has been discovered. To decrease curing temperature and energy consumption, low concentration of curing boosting agents have been added into systems such as benzyl dimethylamine and 2-ethyl-4-methylimidazole^6^. Existence of curing boosting agents has been found to make acid-anhydride derived curing-agents cure epoxy matrices at temperature below 80 °C. Moreover, to notably reduce system viscosity and promote system processability, reactive diluent components have been introduced to dilute the entire adhesive system such as butyl and phenyl glycidyl ethers^7^. Apart from polymer matrix, thermally conductive filler is the other important component of hybrid potting adhesives. Inorganic filler particles such as silica, silicon nitride and alumina have been added to increase heat conductivity and reduce heat-induced expansivity of cured composite potting adhesives^8^.

At present, heat-oxygen induced aging properties of epoxy based hybrid potting adhesives have been widely investigated to evaluate the service life of materials^9^. Those studies are made based on some premises as follows. Epoxy matrix would decompose if enough oxygen radicals are provided. Decomposition of matrix would lead to decline of overall properties of entire cured composite. What’s worse, products from matrix decomposition would further quicken decomposition of matrix. In real environment, concentration of oxygen radicals is rather low. To shorten research period, accelerating-ageing strategy has been applied to explore endurance of systems, based on improvement of strength, frequency or temperature of ageing process^10^.

In the past decade, high overall performances have been found to difficultly achieve in electrical insulation applicable potting adhesive materials, including low dielectric loss, high electric breakdown strength, high mechanical shock strength, high thermal conductivity and high weight stability. To deal with this challenge, in this work, we have designed and prepared a series of epoxy based hybrid potting adhesives filled with inorganic particles in micro-size, through facile physical blend and heat-induced curing process. Silica, alumina and SiC particles in alpha crystalline form were employed as fillers. The most important difference among the three types of fillers lies in their varied band gaps, which has been expected to realize the different dielectric responses (under the applied electric fields) and electrically insulating features (namely electric breakdown strengths) in the cured composites. Two kinds of epoxy resins with different molecular weights were used as matrices. High-performance acid-anhydride type curing agent was utilized. Large band gap of silica filler contributed to high breakdown strength and heat-oxygen-ageing breakdown strength of cured composite adhesive, and relatively high deformation trait of silica particles contributed to high shock strength of cured composite. Silica filler was confirmed to be superior to other two classes of fillers, from the angle of high overall properties of cured composites. At last, high breakdown strength of ~48 MV m^−1^ and high shock strength of ~9950 J m^−2^ were achieved in silica filled cured composite. Therefore, this work might provide a facile route for large-scale fabrication of promising epoxy based composite potting-adhesives used under electrical insulation occasions.

## Experimental

### Materials

Bisphenol-A epoxy resin (JY462) with high molecular weight (high-M_w_) was bought from Guangzhou Aichuan Chemical Co., Ltd. and it was heated to 68 °C to elevate its flowability before utilization. Bisphenol-A epoxy resin (CY221) with low molecular weight (low-M_w_) was gained from Shanghai Kaiping Resin Co., Ltd. Acid anhydride curing-agent (HY91) was achieved from Shanghai Kaiyin Chemical Co., Ltd. and it should be sealed to depress hydrolysis before use. Amine curing-accelerator (0382#) was purchased from Changzhou Jiama Yusheng Chemical Co., Ltd. Alpha-silica particles (average particle size 10 μm, purity >99.5%) were bought from Xinyi Hongrun Quartz Silicon Powder Co., Ltd. Alpha-alumina particles (average grain size 10 μm, purity >99.5%) were obtained from Guangzhou Changbiao Chemical Co., Ltd. Alpha-SiC particles (average particle size 10 μm, purity >99.5%) were purchased from Xi’an Tongxin Semiconductor Accessories Co., Ltd. All of particle-shaped materials should be dried at 110 °C for 5 h to remove surface-absorption water before employment. Besides of special treatments mentioned above, the other materials were directly used as received.

### Fabrication of hybrid potting-adhesives

**Figure 1:**
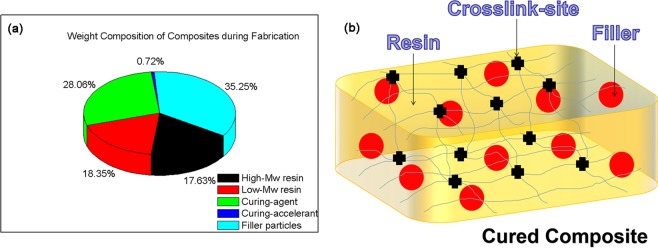
(**a**) Weight composition (formula) of all composite potting adhesives during their fabrication and (**b**) schematic structure of cured composite adhesives with cross-link sites.

All epoxy based hybrid potting adhesives in flowable state were fabricated according to the following formula (see Fig. 1(a)): High-M_w_ resin (JY462, 17.63 wt%), low-M_w_ resin (CY221, 18.35 wt%), curing-agent (HY91, 28.06 wt%), curing-accelerant (0382#, 0.72 wt%) and filler particles (silica, alumina or SiC, 35.25 wt%). Firstly, low-M_w_ resin with a low viscosity was fully added into a metal container, followed by group-by-group incorporating high-M_w_ resin with a high viscosity by vigorous stir, rather than adding high-M_w_ resin all at once. Once all high-M_w_ resin was introduced, the blend was intensively stirred to achieve desirable homogeneous distribution of two resin components. After that, all curing-agent was quickly added followed by strong stir. Moreover, all curing-accelerant was introduced followed by adequate stir. At last, filler particles were added group by group under constant robust stir to achieve favorable high homogeneity in resultant mobile hybrid potting-adhesives. If excessively high viscosity was formed in blend at any stage, the container with the blend should be heated to 68 °C. As-prepared flowable adhesives would be cured or cross-linked via thermal mechanism for following properties measurements.

### Heat-induced curing of mobile composite adhesives

Flowable composite adhesive was slowly poured into a standard mold for shock strength test and a self-made cubic mold for electric breakdown strength measurement respectively, followed by stable heating at 68 °C for 20 min to improve resin flowability, partial removing of inner air under reduced pressure for 6 times and gradual curing at 85 °C for 2 days under atmospheric pressure. Once all the steps above were finished, the cured composite adhesives in solid and cross-link states could be obtained (see Fig. 1(b)). Note that the other property tests of cured composites required no especial molds.

### Tests of heat-oxygen ageing and mass loss ratio of cured composites

Operation steps of measurement of heat-oxygen ageing of cured adhesives were shown as follows. An open oven without reduced pressure was set at 150 °C and further maintained at this temperature. Cured composite sample with its mold was put into that hot oven and aged for 25 days. Because of high temperature and oxygen supply from atmosphere, accelerated heat-oxygen ageing could take place in that adhesive sample. In this work, the influence of heat-oxygen ageing on electric breakdown strength of cured composites would be studied. Moreover, the test of mass loss ratio of cured composites was made as follows. Open oven was set and further maintained at 85 °C, followed by placing cured composite sample (with a small glass beaker as mold) into the oven. Reduced pressure at 0.09 MPa was carried out for interior of that oven. Under high temperature and low pressure, cured sample would slowly release its inner uncrosslinked and volatile substances with low molecule weight, resulting in time-dependent weight loss of sample. By this test, solid content of cured composites could be preliminarily evaluated.

### Characterization

Permittivity and dielectric loss results at 1 kHz were obtained by a HP4284A LCR meter under 1 V bias-voltage. Electric breakdown strength measurements were carried out on a high-voltage electric breakdown instrument (BDJC-100KV). Mechanical shock strength results were gained by a simply supported beam impact testing machine (XJJ-50) based on GB/T 1043-93 standard^11^. Thermal conductivity results at room temperature were achieved on a thermal conductivity tester (DRE-III). Heat-oxygen ageing process and mass loss process were operated by an ordinary air dry oven (WGL-30B). Iron mold for shock strength tests possessed eleven cuboid-shaped fillisters with a size of 1 cm × 0.6 cm × 15 cm. Rubber mold for breakdown strength tests had an internal size of 3 cm × 3 cm × 2.5 cm, equipped with two metal cylinder electrodes with 2 cm length and 28 mm^2^ cross-section area. Electrode distance of 1 mm was maintained. Au electrodes were sputtered onto two surfaces of cured sample by a JEOL JFC-1600 auto fine coater for dielectric properties measurements. Au-sputtering conditions were 20 mA, 10 s and 3 times. Note that two same parallel samples should be prepared and further used for all the tests in this work. For each property datum achieved in this work, four measuring number of times for each sample should be demanded. To obtain the final statistical property results of per-class sample, the evaluating method is stated as follows. For the first parallel sample, the average value of four data determined should be calculated and labeled as *X*_1_. For the second parallel sample, that average value from four results should be achieved and labeled as *X*_2_. As a result, the (*X*_1_ + *X*_2_)/2 value is confirmed to be the final property result of the two samples as one class.

## Results and discussion

### Dielectric properties of cured composites

**Figure 2:**
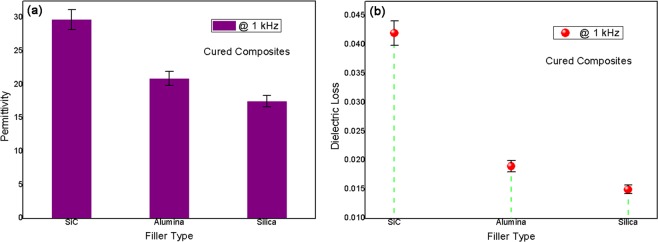
(**a**) Permittivity and (**b**) dielectric loss of cured composites at 1 kHz.

In Fig. 2, dielectric properties of three cured composites at 1 kHz were shown. Based on Fig. 2(a), the permittivity results were ordered as SiC filled sample > alumina one > silica one, ascribed to increase of band gaps of three inorganic fillers^12^. Filler with larger band gap would lead to its lower conductivity under applied field, meaning smaller conductivity difference between filler and epoxy resin matrix. Epoxy matrix had high insulation and rather low conductivity^13^. In this case, weaker interface polarization (Maxwell-Wagner-Sillars polarization)^14^ would be formed between filler and matrix, resulting in lower permittivity of cured composite sample. For an instance, the lowest permittivity of ca. 17 at 1 kHz (still beyond epoxy resins) was obtained in silica filled composite. In Fig. 2(b), dielectric loss results of all cured composites at 1 kHz were achieved. The same changing trend of loss results as above permittivity ones was found, attributed to reduction of interface polarization extent and thus decrease of interface-induced leakage conductance^15^. Rather low dielectric loss of ca. 0.015 at 1 kHz was obtained in silica filled cured composite adhesive. To sum up, silica micro-particles with very large band gap were highly desired to achieve wide electrical insulation application of epoxy based hybrid potting adhesives.

### Electric breakdown properties of cured composites and their aged counterparts

**Figure 3:**
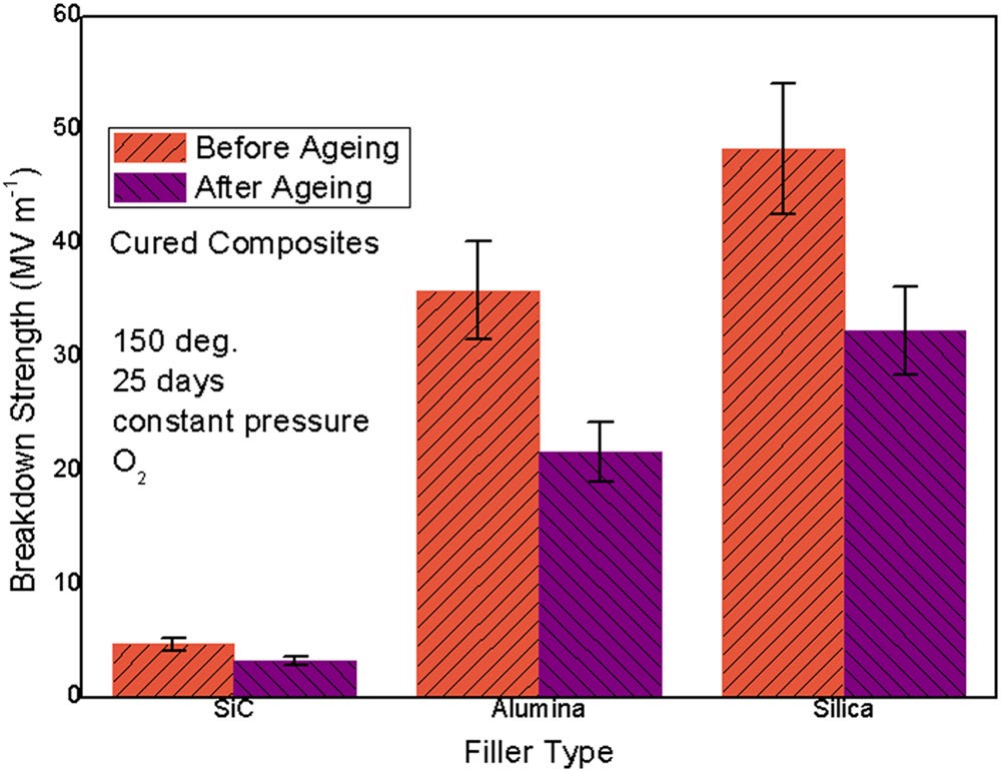
Bkdown reastrength of cured composites and their aged counterparts.

In Fig. 3, breakdown strength results of all cured composites and their aged counterparts were exhibited and compared. As expected, with the increase of filler band gap, breakdown strength of cured composites was improved. On the one hand, filler particles with large band gap meant their low electric conductivity and high breakdown property, contributing to high breakdown performance of cured composite. On the other hand, introducing large-band-gap filler could lead to weak interface polarization between filler and epoxy matrix, suggesting low interface-induced leakage current to obtain high breakdown strength in cured sample^15^. For example, breakdown strength of silica loaded cured composite was measured to be as high as ~48 MV m^−1^ 9.6 times of SiC loaded one (~5 MV m^−1^). The advantage of silica filler among three was further verified. Besides, aged sample was found to always have lower breakdown strength than its corresponding unaged sample, ascribed to local heat-induced chemical decomposition of crosslinked epoxy matrix and thus the motion of low-M_w_ charged substances in aged composite^16^. Fortunately, silica filled aged composite was found to still have the highest breakdown strength (ca. 32 MV m^−1^) among three aged samples, caused by no influence of heat-oxygen ageing on structure and property of inorganic particles^17^. In other words, filler band gap and filler/matrix interface polarization strength would not be altered after ageing. To conclude, favorable high breakdown strength (high electric insulation trait) could be achieved in severely aged composite filled with high-insulation silica. This as-prepared silica filled cured composite adhesive could show a desirable long-term service capacity under high temperature and oxygen atmosphere^18^.

### Mechanical shock properties of cured composites

**Figure 4:**
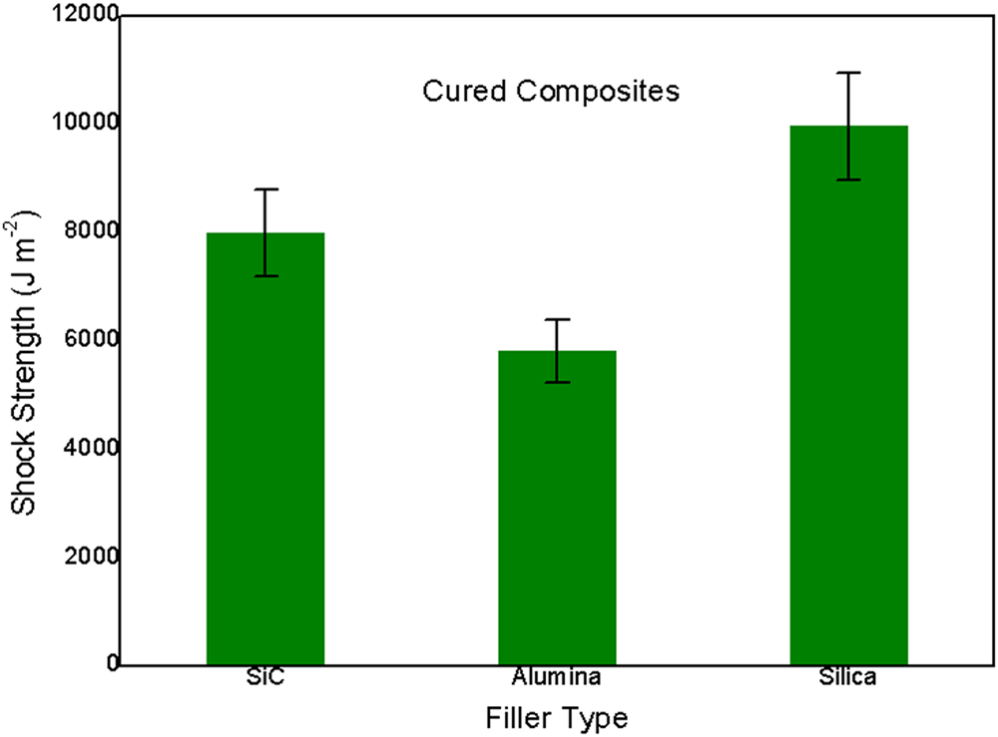
Shock strength of all cured composite adhesives.

Figure 4 displayed shock strength results of three cured composites. Measured shock strength could be ordered as silica loaded sample > SiC loaded one > alumina loaded one, ascribed to promotion of Moh’s hardness of fillers in that order^19^. Filler with low hardness could give rise to high deformation ability of filler, leading to strong shock-energy absorption of filler during shock measurement of cured composite^20^. Due to absorption of shock energy, low applied shock force would not result in fracture of the entire composite. To achieve shock strength of composite at critical fracture state, high shock force should be applied, leading to elevated high shock strength of composite. Obviously, the ‘softest’ silica filler in composite could induce the highest shock strength of ca. 9950 J m^−2^. Herein, silica filled cured composite showed highly desired mechanical property, suggesting its potential application at high stress occasion.

### Heat-conductance performances of cured composites

**Figure 5:**
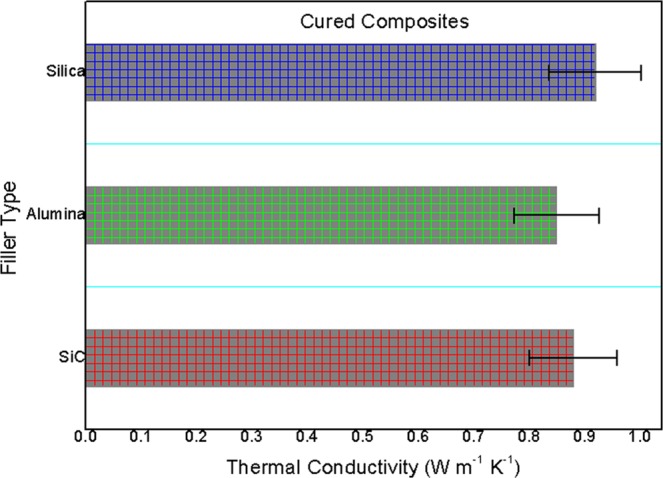
Thermal conductivity of all cured composite adhesives.

Based on Fig. 5, measured thermal conductivity results of all as-prepared cured composites at room temperature were exhibited and compared. As a whole, their heat conductivity results were very close (around 0.9 W m^−1^ K^−1^), ascribed to close inborn thermal conduction feature of three kinds of fillers^21^. Silica filled composite was found to have slight advantage over others based on present testing results. High thermal conductivity was very important for quickly evacuating heat gathered in local regions of composite, avoiding local heat-induced damage and even failure in composite.

### Mass loss features of cured composites at high temperature and low pressure

**Figure 6:**
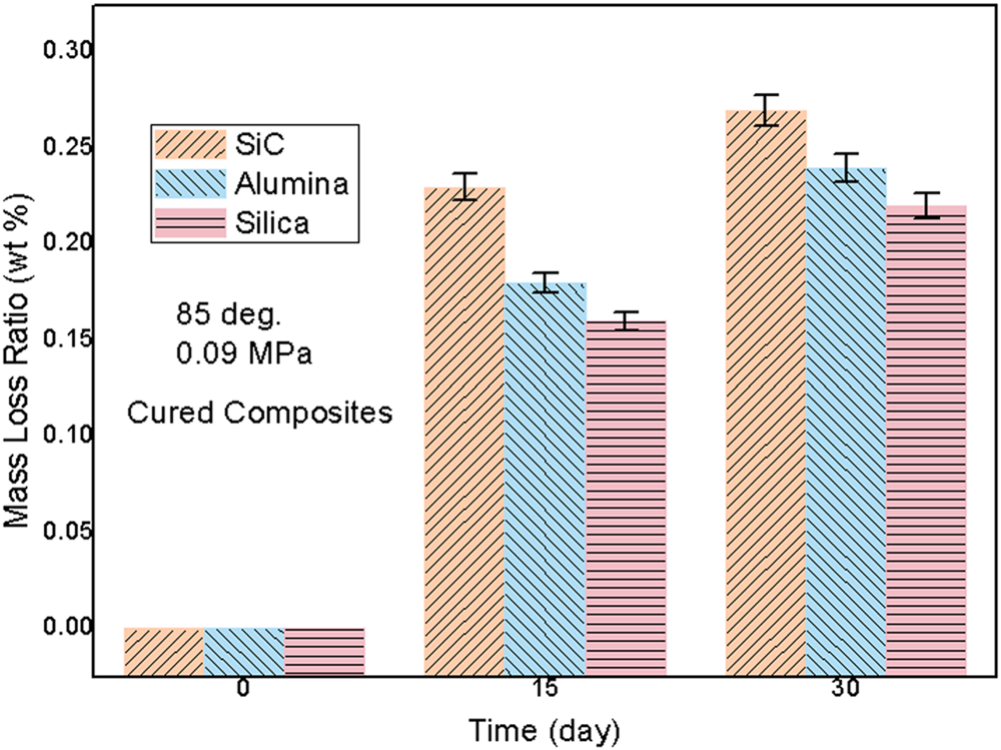
Mass loss ratio of all cured composites as a function of time under high temperature and low pressure.

In Fig. 6, weight loss percentage results of all cured composites as a function of time were obtained, based on high temperature and reduced pressure conditions. In foregoing 15 days, mass loss ratio of composites was found to obviously elevate, attributed to escape of most of residual volatile substances from entire composite. In back 15 days, mass loss ratio of them was observed to increase by a small margin, ascribed to release of a spot of volatile molecules remained in the composite. At 30 days, mass loss ratios of all composites were confirmed to be lower than 0.3 wt%, showing extremely low concentration of unstable components in those composites. That is to say, desirable high solid content was realized in all cured composites. Besides, the highest weight-stability was found in silica filled cured sample, further verifying superiority of silica filler to other fillers. In Table S1, the over-all properties of the cured neat epoxy matrix material were exhibited.

## Conclusion

In this work, inorganic micro-particles filled epoxy based composite potting adhesives with high over-all properties were designed and prepared for electrical insulation application. Cheap and available silica, alumina and SiC particles in alpha crystalline form without surface modification were employed as fillers, respectively. Two classes of epoxy resins with high and low molecular weights were utilized as matrices at the same time. Heat induced curing was operated onto mobile hybrid adhesives fabricated according to optimized formula. Based on various property measurements, silica filled cured composite showed highly desired comprehensive performances including dielectric, breakdown, mechanical, thermal and mass stability properties. To conclude, the low permittivity, low dielectric loss, high electric breakdown strength, high ageing breakdown strength, high shock strength, high thermal conductivity and low weight loss percentage were obtained in micro-silica loaded composite, leading to its promising application in electrical insulation occasion. Large band gap and relatively high deformation ability of silica particles could contribute to favorable breakdown and mechanical properties of composite. Silica filled cured composite exhibited a breakdown strength of ca. 48 MV m^−1^ and shock strength of ca. 9950 J m^−2^. This work might open up the door to large-scale fabrication of high-performance epoxy composite potting-adhesives.

## Supplementary information

Supplementary information.
