# Supplementary material for: High-performance ceramic/epoxy composite adhesives enabled by rational ceramic bandgaps
Source: Sci Rep. 2020 Jan 16;10:484. doi: 10.1038/s41598-019-57074-7 (PMC6965657; doi:10.1038/s41598-019-57074-7)
Supplement: Supplementary file 1 — Supplementary information. [file 41598_2019_57074_MOESM1_ESM.doc]

**Supporting Information for Publication**

High-performance ceramic/epoxy composite adhesives enabled by rational ceramic bandgaps

Jianbing Hu*

The thermo-electro-mechanical properties of the neat epoxy matrix material were exhibited in Table S1.

**Table S1**. Over-all properties of neat epoxy matrix material.

| Permittivity (@1kHz) | Dielectric loss (@1kHz) | Breakdown strength before ageing (MV m^-1^) | Breakdown strength after ageing (MV m^-1^) | Shock strength (J m^-2^) | Thermal conductivity (W m^-1^ K^-1^) | Mass loss ratio at 0 day (wt %) | Mass loss ratio at 15 days (wt %) | Mass loss ratio at 30 days (wt %) |
| --- | --- | --- | --- | --- | --- | --- | --- | --- |
| 3.45±0.10 | 0.0038±0.0001 | 77.42±6.10 | 60.18±4.73 | 14980±1650 | 0.213±0.026 | 0.000±0.000 | 0.251±0.012 | 0.297±0.015 |
